# Microwave‐assisted extraction of bioactive compounds from Sarawak *Liberica* sp. coffee pulp: Statistical optimization and comparison with conventional methods

**DOI:** 10.1002/fsn3.3494

**Published:** 2023-06-11

**Authors:** Joel Ching Jue Wong, Elexson Nillian

**Affiliations:** ^1^ Faculty of Resource Science and Technology University Malaysia Sarawak Kota Samarahan Sarawak Malaysia

**Keywords:** bioactive compounds, *Coffea liberica*, microwave‐assisted extraction, response surface methodology

## Abstract

*Coffea liberica*, commonly known as *Liberica* coffee, is a kind of coffee that originated in Liberia, a West African country. It is considered a less‐known coffee bean variety, which accounts for less than 2% of commercially produced coffee worldwide. In this study, the influences of optimization of microwave‐assisted extraction (MAE) on the total phenolic content (TPC), total flavonoid content (TFC), and total carbohydrate content (TCC) of bioactive compounds extracted from Sarawak *Liberica* sp. coffee pulp were studied. Response surface methodology was adopted with a face‐centered central composite design to generate 34 responses by taking three microwave parameters into consideration, microwave power (watt), time of irradiation (second), and solvent‐to‐feed ratio as independent variables. As a result, the findings revealed that optimum extraction conditions were conducted as follows: microwave power of 700 W, time of irradiation of 180 s, and solvent‐to‐feed ratio of 86.644:1. While under optimal extraction conditions, MAE outperformed conventional maceration extraction in terms of extraction efficiency and had no significant difference (*p* < .05) with Soxhlet extraction on the extraction of TPC (12.94 ± 2.25 mg GAE/g), TFC (9.84 ± 0.38 mg QE/g), and TCC (876.50 ± 64.15 mg GE/g). Present work advances the usage of Sarawak *Liberica* sp. coffee for the development of functional products and aids in reducing environmental pollution by utilization of coffee pulp waste.

## Graphical abstract

This study analyzed the extraction of bioactive compounds from Sarawak *Liberica* sp. coffee pulp using microwave‐assisted extraction (MAE) and response surface methodology. The optimal extraction conditions were determined to be 700 W, 180 s, and a solvent‐to‐feed ratio of 86.644:1. The results showed that MAE was more efficient than conventional maceration extraction and had no significant difference in terms of extraction efficiency compared to Soxhlet extraction.

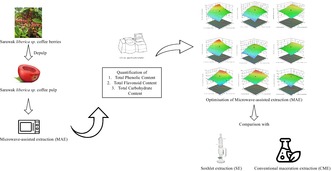

## 1 INTRODUCTION

One of the most popular beverages in the world is coffee, which is also a key commercial food item. Arabica (80%) and Robusta (20%) are the two most widely grown and traded varieties of coffee, whereas Liberica accounts for less than 1% of global production (Ismail et al., 2022). In Malaysia, however, Arabica is only very rarely grown in highland areas, with Liberica (73%) and Robusta (27%) being the two most popular coffee kinds. According to Azmil (1991), Robusta and Liberica coffee may be successfully grown in Malaysia because of its ideal growing temperature range of 18–28°C (maximum at 34°C). Nevertheless, Arabica can only be cultivated in altitude regions, such as the Cameron Highlands in Pahang, where temperatures must be below 23°C.

Coffee manufacturing generates a variety of by‐products, including wasted coffee grounds, coffee wastewater, coffee pulp, coffee husks, coffee silver skin, and coffee parchment (Reichembach & Petkowicz, 2020). Coffee pulp makes up most of the solid waste produced during the wet processing of coffee. Studies on coffee pulp have confirmed coffee pulp to be abundant in health‐promoting nutrients such as fiber, proteins, carbohydrates, minerals (especially potassium), tannins, polyphenols, and caffeine (Murthy & Madhava Naidu, 2012). In addition, Sarawak *Liberica* sp. coffee pulp has been found to have high levels of phenolic and flavonoid compounds, reducing sugar, and a higher level of radical scavenging activity (Nillian et al., 2020), indicating its potential as a natural source for obtaining bioactive compounds of interest and further developing into functional food. For example, antioxidant‐rich food products such as energy bars, cereal bars, or snack bars can be developed from the extracted phenolic and flavonoid compounds. Besides, it can also be used as an ingredient in prebiotic supplements, such as capsules or powders, due to its high level of reducing sugars, which promote the growth of beneficial bacteria in the gut. However, coffee pulp is said to harm the ecosystem by contaminating lakes and rivers close to coffee processing facilities. If marketable by‐products are extracted from the coffee pulp in large‐scale commercial secondary processing installations using a technology that is beneficial to the environment, this issue might turn into an opportunity (Manasa et al., 2021). Therefore, it may be better to exploit the coffee pulp as a source for extracting bioactive compounds.

To isolate bioactive ingredients from natural sources, extraction is the initial and most crucial stage. The knowledge of effective bioactive compound extraction from Liberica needs to be improved. It has been discovered that traditional extraction methods, including maceration, infusion, and superficial extraction take a long time, cost a lot to operate, and provide minimal output (Fang et al., 2011). However, Ince et al. (2012) found that microwave‐assisted extraction (MAE) may be used for the substitution of traditional extraction methods and increase bioactive compounds' yield while also speeding up the extraction process and using less solvent. One of the cutting‐edge extraction methods, MAE, has been widely used to separate, evaluate, and quantify bioactive components. In MAE, solvents are heated when they encounter solid or liquid samples (or heated samples, such as fresh tissues), facilitating the partition of chemicals relevant to the sample into the solvent. According to published research, several variables, for example, the type of solvent, power, microwave irradiation time, sample‐to‐solvent ratio, and matrix type, affect MAE yields (Mandal et al., 2007). In light of the fact that coffee pulp may be used to obtain bioactive compounds, it will be useful to determine the experimental conditions for extracting bioactive compounds with the highest yield using MAE.

To achieve maximum extraction efficiency, the influence of MAE process factors and their interactions were assessed using a response surface methodology (RSM). With several experiment runs, this method predicts the most compelling circumstances while allowing simultaneous optimization of all factors. Therefore, this study aims to evaluate the optimization of MAE conditions to obtain the maximum yield of total phenolic, flavonoid, and carbohydrate content from Sarawak *Liberica* sp. coffee pulp and to compare the effectiveness of MAE with conventional maceration extraction (CME) and Soxhlet extraction (SE).

## 2 MATERIALS AND METHODS

### 2.1 Materials

The Sarawak *Liberica* sp. coffee pulp was used in this study. The coffee pulp samples were provided by RekaJaya Plantation Sdn. Bhd. in Kuching, Sarawak and were sent to the laboratory and refrigerated at 4°C prior to investigation. Unless otherwise mentioned, all chemicals and reagents involved in the study were of analytical grade.

### 2.2 Sample preparation of Sarawak *Liberica* sp. coffee pulp fibrous powder

Sarawak *Liberica* sp. coffee pulp was made into powder form according to the guidelines recommended by Larrauri (1999). Firstly, coffee pulp samples were collected and cleaned with distilled water to get rid of the dirt. The weight of the pulp samples was determined. The pulp was then processed using a wet‐milling method with a 1 g of pulp to 1 ml of water ratio. This process was performed using a food blender (Sharp blender EM131BK). A cotton cloth filter was used to separate the juice and pomace from the pulp. After that, the pomace was weighed and measured. The pomace was then washed in water for 5 min. Next, cotton cloth filter was used to filter the pomace, and the pomace was dried in an oven dryer (Drying oven FCD‐3000) for 18 h at 50°C. After drying, the pomace was ground into powder form using the food blender (Sharp blender EM131BK). Then, the powder was sieved to pass through a standard 150‐μm sieve to obtain Sarawak *Liberica* sp. coffee pulp fibrous powder. The powder was kept at −20°C prior to analysis.

### 2.3 Experimental design

The method used for extraction and optimization was MAE under different parameters and levels as shown in Table 1. For comparison, CME and SE were also conducted. In this study, RSM with face‐centered central composite design (CCD) was applied. CCD was selected to investigate the effects of multiple variables on the extraction of bioactive compounds and to determine the optimal conditions for maximizing the extraction of bioactive compounds from Sarawak *Liberica* sp. coffee pulp. CCD allows the investigation of both linear and quadratic effects of the input variables on the response and provides insight into the curvature of the response surface, allowing for the identification of optimum conditions for maximum response. Three microwave parameters: microwave power (watt, *X*_1_), time of irradiation (seconds, *X*_2_), and solvent‐to‐feed ratio (*X*_3_) were examined for their impacts on three response variables: total phenolic content (TPC, mg GAE/g extract, *Y*_1_), total flavonoid content (TFC, mg QE/g extract, *Y*_2_), and total carbohydrate content (TCC, mg GE/g extract, *Y*_3_). According to the minimum and maximum readings of the parameters selected based on literature data and early experimental findings, each parameter was examined at two levels: high (+1) and low (−1) levels. Methanol was used as the solvent for the extraction of bioactive compounds due to its high polarity, which allows it to effectively dissolve and extract a wide range of polar and semi‐polar compounds from the sample matrix. Design‐Expert® Software (Version 13.0; Stat‐Ease, Inc.) was used to carry out the experimental design.

**TABLE 1:** Microwave parameters and their levels used in CCD.

Microwave parameters	Unit	Notation	Levels	
			Low (−1)	High (+1)
Microwave power	Watt	*X*_1_	119	700
Time of irradiation	Second	*X*_2_	30	180
Solvent‐to‐feed ratio	‐	*X*_3_	10:1	100:1

Abbreviation: CCD, central composite design.

### 2.4 Extraction procedures

#### 2.4.1 Microwave‐assisted extraction

Microwave‐assisted extraction was performed using a microwave oven (Elba EMO‐2072SV) operating at 700 W output power, 2450 MHz frequency, and 20 L capacity. Sarawak *Liberica* sp. coffee pulp fibrous powder was mixed with methanol (CH_3_OH, 99% v/v) to obtain the ratio as indicated in Table 2. Then, extraction was carried out at the respective settings of microwave power and irradiation time (Table 2). Thereafter, the mixture was centrifuged twice at 5000 rpm, 4°C for 10 min by a centrifuge machine (Hanil Scientific, Inc.; Supra R22). Next, the supernatant was collected and added with 1 mL of trichloroacetic acid (C_2_HCl_3_O_2_, 10% w/v) for the precipitation of protein (Novák & Havlíček, 2016). The precipitate was removed and the supernatant was collected and dialyzed against distilled water for 12 h using a dialysis bag (molecular weight cut‐off, MWCO 500 Da) to eliminate the unwanted low‐molecular‐weight compounds. Liquid extract that had not been dialyzed was collected and kept at 4°C prior to investigation.

**TABLE 2:** Design matrix of central composite design (CCD) with TPC, TFC, and TCC as responses.

Run	Microwave parameters^a^			Responses^b^		
	*X*_1_	*X*_2_	*X*_3_	*Y*_1_	*Y*_2_	*Y*_3_
1	409.5	120	55	8.40 ± 0.62	6.37 ± 6.69	555.81 ± 24.41
2	119	60	100	3.41 ± 0.69	0.85 ± 15.92	271.66 ± 174.81
3	700	60	10	3.87 ± 0.10	2.41 ± 1.23	307.63 ± 8.23
4	119	180	100	4.63 ± 0.33	1.98 ± 15.55	323.79 ± 48.99
5	119	180	10	3.21 ± 0.000	1.42 ± 8.43	320.13 ± 22.92
6	700	180	100	13.08 ± 1.07	9.76 ± 10.60	836.92 ± 16.54
7	119	60	10	1.49 ± 0.08	0.82 ± 2.92	188.42 ± 33.19
8	700	180	10	9.37 ± 0.40	9.62 ± 2.96	750.94 ± 23.73
9	409.5	120	55	8.25 ± 0.13	5.52 ± 3.29	612.82 ± 42.18
10	700	60	100	7.54 ± 0.13	6.37 ± 0.51	618.31 ± 62.92
11	119	180	100	4.79 ± 0.37	2.26 ± 12.41	330.19 ± 3.17
12	700	180	100	13.289 ± 1.20	10.47 ± 5.41	819.54 ± 24.59
13	700	180	10	9.72 ± 0.75	8.77 ± 3.16	780.21 ± 166.04
14	409.5	120	55	8.40 ± 0.23	5.80 ± 3.30	570.75 ± 50.36
15	409.5	120	55	8.55 ± 0.31	6.37 ± 6.26	610.99 ± 51.20
16	700	60	10	4.12 ± 0.26	2.69 ± 0.51	328.36 ± 11.46
17	119	60	100	3.00 ± 0.20	1.56 ± 14.08	285.38 ± 11.13
18	700	60	100	6.92 ± 0.29	4.95 ± 7.74	638.43 ± 57.78
19	119	180	10	3.56 ± 0.94	2.26 ± 4.90	314.41 ± 14.13
20	119	60	10	0.71 ± 0.18	0.14 ± 11.23	213.12 ± 12.71
21	409.5	180	55	9.98 ± 0.22	7.92 ± 3.85	485.69 ± 35.99
22	700	120	55	8.50 ± 0.44	5.52 ± 1.50	720.57 ± 39.62
23	409.5	120	100	7.84 ± 0.05	3.82 ± 1.21	640.26 ± 84.91
24	409.5	60	55	6.31 ± 0.14	5.24 ± 20.76	278.97 ± 3.30
25	409.5	120	10	5.60 ± 0.28	4.39 ± 12.60	446.36 ± 22.85
26	409.5	120	55	8.25 ± 1.32	6.65 ± 2.62	578.98 ± 30.98
27	409.5	120	55	8.10 ± 0.52	5.52 ± 4.34	594.53 ± 11.09
28	409.5	120	10	5.65 ± 1.02	3.82 ± 0.79	414.34 ± 14.37
29	409.5	60	55	6.21 ± 0.32	4.81 ± 7.18	442.70 ± 43.71
30	119	120	55	2.39 ± 0.09	2.12 ± 2.86	375.87 ± 31.28
31	409.5	180	55	10.84 ± 0.14	7.08 ± 2.50	497.58 ± 54.81
32	119	120	55	2.14 ± 0.67	1.56 ± 1.21	442.51 ± 36.44
33	700	120	55	7.54 ± 0.67	5.94 ± 3.80	803.98 ± 3.30
34	409.5	120	100	7.03 ± 0.18	5.52 ± 0.14	651.24 ± 12.58

Abbreviations: TCC, total carbohydrate content; TFC, total flavonoid content; TPC, total phenolic content.

^a^ Parameters: *X*_1_, microwave power (watt); *X*_2_, time of irradiation (second); *X*_3_, solvent‐to‐feed ratio.

^b^ Experimental results of the responses: *Y*_1_, total phenolic content (mg GAE/g); *Y*_2_, total flavonoid content (mg QE/g); *Y*_3_, total carbohydrate content (mg GE/g).

#### 2.4.2 Conventional maceration extraction

Conventional maceration extraction was carried out at room temperature in the following manner: 5 g of Sarawak *Liberica* sp. coffee pulp fibrous powder was mixed with 350 mL of methanol (CH_3_OH, 99% v/v). Then, the mixture was kept at room temperature overnight on a shaker (Shaker JEIO TECH SK‐71). Thereafter, the mixture was centrifuged twice at 5000 rpm, 4°C for 10 min by a centrifuge machine (Hanil Scientific, Inc.; Supra R22). Next, the supernatant was collected and added with 1 mL of trichloroacetic acid (C_2_HCl_3_O_2_, 10% w/v) for the precipitation of protein. The precipitate was removed and the supernatant was collected and dialyzed against distilled water for 12 h using a dialysis bag (molecular weight cut‐off, MWCO 500 Da) to eliminate the unwanted low‐molecular‐weight compounds. Liquid extract that had not been dialyzed was collected and kept at 4°C prior to investigation.

#### 2.4.3 Soxhlet extraction

A measured amount of Sarawak *Liberica* sp. coffee pulp fibrous powder (5 g) was placed in a cellulose thimble of the Soxhlet extractor. An amount of 350‐mL methanol (CH_3_OH, 99% v/v) was added into a round bottom flask of the Soxhlet extractor. Then, the Soxhlet extractor was set up and methanol was heated using the isomantle heater. The extraction time was 10 h and the temperature was set at 65°C. Thereafter, the mixture was centrifuged twice at 5000 rpm, 4°C for 10 min by a centrifuge machine (Hanil Scientific, Inc.; Supra R22). Next, the supernatant was collected and added with 1 mL of trichloroacetic acid (C_2_HCl_3_O_2_, 10% w/v) for the precipitation of protein. The precipitate was removed and the supernatant was collected and dialyzed against distilled water for 12 h using a dialysis bag (molecular weight cut‐off, MWCO 500 Da) to eliminate the unwanted low‐molecular‐weight compounds. Liquid extract that had not been dialyzed was collected and kept at 4°C prior to investigation.

### 2.5 Phytochemical analysis

#### 2.5.1 Total phenolic content

Total phenolic content of the extract obtained from MAE, CME, and SE was investigated following the method developed by Velioglu et al. (1998) with some modifications. For sample preparation, liquid extract was dried in a fume hood (Köttermann GmbH fume hood) and resuspended with methanol (CH_3_OH, 99% v/v) to obtain a final concentration of 1 mg/mL. Briefly, 0.2 mL of the sample was mixed with 0.5 mL of stock Folin–Ciocalteu's reagent (10% w/v). Next, 0.8 mL of bicarbonate (NaHCO_3_, 7.5% w/v) solution was added to the mixture. The mixture was vortexed and allowed to stand for 30 min at room temperature with continual shaking. The absorbance of the mixture was obtained using a Ultraviolet–visible spectrophotometer (Shimadzu UV‐1900i) at 765 nm. A calibration curve (absorbance vs. concentration) was constructed using gallic acid solution (0.1–0.5 mg/mL in methanol [CH_3_OH, 99% v/v]) as a standard. The results were expressed as milligram of gallic acid equivalent per gram of dry extract (mg GAE/g extract).

#### 2.5.2 Total flavonoid content

Total flavonoid content of the extract obtained from MAE, CME, and SE was measured using the aluminum chloride colorimetric method as reported by Marinova et al. (2005) with slight modification. Before the test, liquid extract was dried in a fume hood (Köttermann GmbH fume hood) and resuspended with methanol (CH_3_OH, 99% v/v) to obtain a final concentration of 1 mg/mL. Firstly, 0.4 mL of the sample was mixed with 0.3 mL of sodium nitrite (NaNO_2_, 5% w/v). The mixture was let to stand for 5 min. Then, 0.3 mL of aluminum chloride (AlCl_3_, 10% w/v) was added to the mixture and shaken well. The mixture was allowed to stand for another 5 min. Thereafter, 2.0 mL of sodium hydroxide (NaOH, 1 M) and 4.0 mL of distilled water were added to the mixture, and the test tube was vortexed before the measurement of absorbance at 510 nm using Ultraviolet–visible spectrophotometer (Shimadzu UV‐1900i). A calibration curve (absorbance vs. concentration) was made using quercetin (0.2–1.0 mg/mL in 99% [v/v] methanol). The results were presented as milligram of quercetin equivalent per gram of dry extract (mg QE/g extract).

#### 2.5.3 Total carbohydrate content

Total carbohydrate content of the extract obtained from MAE, CME, and SE was tested by phenol‐sulfuric method as described by Dubois et al. (1956). At first, liquid extract was dried in a fume hood (Köttermann GmbH fume hood) and resuspended with methanol (CH_3_OH, 99% v/v) to obtain a final concentration of 1 mg/mL. Briefly, 1 mL of phenol solution (C_6_H_6_O, 5% v/v) and 5‐mL concentrated sulfuric acid (H_2_SO_4_, 97% v/v) were added to 1 mL of the sample. The mixture was vortexed and incubated in a water bath (Memmert Waterbath WNB 14) at 30°C for 30 min. The absorbance readings were determined by a Ultraviolet–visible spectrophotometer (Shimadzu UV‐1900i) at 490 nm. d‐glucose (0.02–0.10 mg/mL) was used as a standard for calibration of a standard curve (absorbance vs. concentration). The results were expressed as milligram of d‐glucose equivalent per gram of dry extract (mg GE/g extract).

### 2.6 Statistical analysis

Experiments were done in triplicate and data were presented as mean ± standard deviation. SPSS software (version 23; IBM Corporation) was used to carry out the statistical analysis. Analysis of variance (ANOVA)'s Tukey test, Kruskal–Wallis test, and independent t‐test were applied to determine the significant difference among the means at 95% confidence interval (*p* < .05).

## 3 RESULTS AND DISCUSSION

### 3.1 Experimental outcome of design matrix

A total of 34 experimental runs were carried out at random using the levels described in Table 1 and the conditions are shown in Table 2, which included 14 various combinations of experiments conducted in duplicate and six center points, as well as the experimental results of response of the study.

### 3.2 Model fitting

In the present study, a face‐centered CCD was selected to investigate the influences and interactions of the MAE parameters (microwave power, time of irradiation, and solvent‐to‐feed ratio) on the TPC, TFC, and TCC of the bioactive compounds from Sarawak *Liberica* sp. coffee pulp. The experimental results for TPC, TFC, and TCC are depicted in Table 2. The data demonstrated a significant dependency on the extraction conditions, indicating that the extraction process should be optimized.

The ANOVA results are presented in Table 3. Mathematical models were developed so that the experimental data would achieve a significant fit and find the optimal operating parameters to obtain the best‐measured responses. Greater *F*‐values and smaller *p*‐values indicate more significant model terms; therefore, based on the ANOVA results in Table 3, these results confirmed the significance of the models. The *p*‐values of “lack of fit” test of TPC, TFC, and TCC models were not significant since the values were greater than 0.05. An insignificant “lack of fit” represents an indication of model's good predictability. The results demonstrated the accuracy and predictability of each model.

**TABLE 3:** Analysis of variance (ANOVA) of quadratic model for TPC, TFC, and TCC.

	Source	Sum of squares	df	Mean square	*F*‐value	*p*‐Value	Remark
TPC	Block	2.04	2	1.02			
	Model	326.38	9	36.26	155.60	<.0001	Significant
	*X*_1_‐Microwave power	154.62	1	154.62	663.44	<.0001	
	*X*_2_‐Time of irradiation	75.57	1	75.57	324.24	<.0001	
	*X*_3_‐Solvent‐to‐feed ratio	29.37	1	29.37	126.00	<.0001	
	*X*_1_ *X*_2_	14.91	1	14.91	63.96	<.0001	
	*X*_1_ *X*_3_	2.97	1	2.97	12.73	.0017	
	*X*_2_ *X*_3_	0.0348	1	0.0348	0.1492	.7030	
	$X_{1}^{2}$	33.99	1	33.99	145.84	<.0001	
	$X_{2}^{2}$	3.54	1	3.54	15.19	.0008	
	$X_{3}^{2}$	4.64	1	4.64	19.90	.0002	
	Residual	5.13	22	0.2331			
	Lack of fit	3.11	13	0.2395	1.07	.4721	Not significant
	Pure error	2.01	9	0.2238			
	Cor error	333.55	33				
TFC	Block	2.08	2	1.04			
	Model	239.37	9	26.60	55.15	<.0001	Significant
	*X*_1_‐Microwave power	132.80	1	132.80	275.36	<.0001	
	*X*_2_‐Time of irradiation	49.04	1	49.04	101.68	<.0001	
	*X*_3_‐Solvent‐to‐feed ratio	6.27	1	6.27	13.01	.0016	
	*X*_1_ *X*_2_	19.50	1	19.50	40.43	<.0001	
	*X*_1_ *X*_3_	2.29	1	2.29	4.75	.0402	
	*X*_2_ *X*_3_	1.73	1	1.73	3.59	.0714	
	$X_{1}^{2}$	14.78	1	14.78	30.65	<.0001	
	$X_{2}^{2}$	3.71	1	3.71	7.69	.0111	
	$X_{3}^{2}$	6.24	1	6.24	12.93	.0016	
	Residual	10.61	22	0.4823			
	Lack of fit	7.23	13	0.5565	1.48	.2802	Not significant
	Pure error	3.38	9	0.3751			
	Cor error	252.05	33				
TCC	Block	15632.83	2	7816.42			
	Model	1.114E + 06	9	1.238E + 05	60.92	<.0001	Significant
	*X*_1_‐Microwave Power	6.264E + 05	1	6.264E + 05	308.32	<.0001	
	*X*_2_‐Time of Irradiation	1.779E + 05	1	1.779E + 05	87.58	<.0001	
	*X*_3_‐Solvent‐to‐feed Ratio	91368.16	1	91368.16	44.97	<.0001	
	*X*_1_ *X*_2_	58189.38	1	58189.38	28.64	<.0001	
	*X*_1_ *X*_3_	20386.34	1	20386.34	10.03	.0045	
	*X*_2_ *X*_3_	24924.12	1	24924.12	12.27	.0020	
	$X_{1}^{2}$	2971.73	1	2971.73	1.46	.2393	
	$X_{2}^{2}$	91599.19	1	91599.19	45.09	<.0001	
	$X_{3}^{2}$	2732.23	1	2732.23	1.34	.2586	
	Residual	44694.34	22	2031.56			
	Lack of fit	22393.77	13	1722.60	0.6952	.7330	Not significant
	Pure error	22300.57	9	2477.84			
	Cor error	1.174E + 06	33				

Abbreviations: TCC, total carbohydrate content; TFC, total flavonoid content; TPC, total phenolic content.

As indicated by fit statistics in Table 4, the level of correspondence between experimental and predicted values of TPC, TFC, and TCC was stated by *R* ^2^ values (TPC—.9845, TFC—.9576, and TCC—.9614). It indicates that the variables in the design matrix explain the variation occurring in TPC, TFC, and TCC by 98.45%, 95.76%, and 96.14%, respectively. The adjusted *R* ^2^ and predicted *R* ^2^ values were .9782 and .9656 for TPC, .9402 and .8985 for TFC, and .9614 and .9114 for TCC. The fact that the differences between adjusted *R* ^2^ and predicted *R* ^2^ of the three models were all less than .2 implies that the predicted and actual experimental results were in reasonable agreement with each other. Other than that, the low coefficient of variation values (CV = 7.40%, 14.69%, and 8.99% for TPC, TFC, and TCC, respectively) indicates that the experimental results of the fitted models were reliable and reproducible. It was recommended that the models had a decent simulation of the extraction experiment based on the findings.

**TABLE 4:** Fit statistics.

Model	Std. dev.	Mean	C.V.%	*R* ^2^	Adjusted *R* ^2^	Predicted *R* ^2^	Ade. Pre.
TPC	0.4828	6.52	7.40	.9845	.9782	.9656	41.4528
TFC	0.6945	4.73	14.69	.9576	.9402	.8985	21.8381
TCC	45.07	501.51	8.99	.9614	.9456	.9114	25.7030

Abbreviations: TCC, total carbohydrate content; TFC, total flavonoid content; TPC, total phenolic content.

Predictions about the tested responses can be made using the following coded equations (Equations (1), (2), (3)). By contrasting the factor coefficients, the coded equation may be used to determine the relative significance of the factors. A positive effect is indicated by a positive coefficient, and vice versa.

Equation (1) shows that TPC obtained increases as all the parameters (*X*_1_, *X*_2_, and *X*_3_), interaction effect *X*_1_ *X*_2_ and *X*_1_ *X*_3_, and quadratic terms $X_{2}^{2}$ increase, and it increases as the quadratic terms $X_{1}^{2}$ and $X_{3}^{2}$ decrease. TPC obtained has a positive relation with microwave power (*X*_1_), time of irradiation (*X*_2_), solvent‐to‐feed ratio (*X*_3_), interaction effect (microwave power and time of irradiation, *X*_1_ *X*_2_), interaction effect (microwave power and solvent‐to‐feed ratio, *X*_1_ *X*_3_), and the quadratic effect $X_{2}^{2}$, whereas it has a negative relation with the quadratic effects $X_{1}^{2}$ and $X_{3}^{2}$. There was no significant interactive effect (*p* = .7030 [>.05]) of *X*_2_ *X*_3_ on TPC. This indicates that the TPC of the bioactive compounds from Sarawak *Liberica* sp. coffee pulp was not affected by the interaction effect between time of irradiation and solvent‐to‐feed ratio. The factor coefficient of *X*_1_ (2.78) has the highest number among all the parameters. Thus, microwave power had the most significant effect on TPC.

Similar to TPC, Equation (2) shows that TFC obtained increases as all the parameters (*X*_1_, *X*_2_, and *X*_3_), interaction effect *X*_1_ *X*_2_ and *X*_1_ *X*_3_, and quadratic terms $X_{2}^{2}$ increase, and it increases as the quadratic terms $X_{1}^{2}$ and $X_{3}^{2}$ decrease. TFC obtained has a positive relation with microwave power (*X*_1_), time of irradiation (*X*_2_), solvent‐to‐feed ratio (*X*_3_), interaction effect (microwave power and time of irradiation, *X*_1_ *X*_2_), interaction effect (microwave power and solvent‐to‐feed ratio, *X*_1_ *X*_3_), and the quadratic effect $X_{2}^{2}$, whereas it has a negative relation with the quadratic effects $X_{1}^{2}$ and $X_{3}^{2}$. There was no significant interactive effect (*p* = .7030 [>.05]) of *X*_2_ *X*_3_ on TFC. This indicates that the TFC of the bioactive compounds from Sarawak *Liberica* sp. coffee pulp was not affected by the interaction effect between time of irradiation and solvent‐to‐feed ratio. The factor coefficient of *X*_1_ (2.58) has the highest number among all the parameters. Therefore, the most significant effect on TFC was caused by microwave power.

As indicated in Equation (3), the TCC obtained increases as all the parameters (*X*_1_, *X*_2_, and *X*_3_) and the interaction effect *X*_1_ *X*_2_ and *X*_1_ *X*_3_ increase, and it increases as the interaction effect *X*_2_ *X*_3_ and the quadratic terms $X_{2}^{2}$ decrease. TCC obtained has a positive relation with microwave power (*X*_1_), time of irradiation (*X*_2_), solvent‐to‐feed ratio (*X*_3_), interaction effect (microwave power and time of irradiation, *X*_1_ *X*_2_), and interaction effect (microwave power and solvent‐to‐feed ratio, *X*_1_ *X*_3_), whereas it has a negative relation with the interaction effect (time of irradiation and solvent‐to‐feed ratio, *X*_2_ *X*_3_) and quadratic effects $X_{2}^{2}$. There were no significant quadratic effects of $X_{1}^{2}$ (*p* = .2393 [>.05]) and $X_{3}^{2}$ (*p* = .2586 [>.05]) on TCC. This indicates that the TCC of the bioactive compounds from Sarawak *Liberica* sp. coffee pulp was not affected by the quadratic effects of $X_{1}^{2}$ and $X_{3}^{2}$. The factor coefficient of *X*_1_ (176.97) has the highest number among all the parameters. Hence, microwave power was the most significant influencing factor on TCC.

The mathematical models in terms of coded factors for the tested responses can be represented as follows:

(1) $$\begin{array}{c} Y_{\mathrm{TPC}}=8.20+2.78X_{1}+1.94X_{2}+1.21X_{3}+0.9653X_{1}X_{2}+0.4306X_{1}X_{3}\\ -0.0466X_{2}X_{3}-2.60X_{1}^{2}+0.8404X_{2}^{2}-0.9619X_{3}^{2} \end{array}$$

(2) $$\begin{array}{c} Y_{\mathrm{TFC}}=5.93+2.58X_{1}+1.57X_{2}+0.5601X_{3}+1.10X_{1}X_{2}\\ +0.3786X_{1}X_{3}-0.3289X_{2}X_{3}-1.72X_{1}^{2}+0.8603X_{2}^{2}-1.12X_{3}^{2} \end{array}$$

(3) $$\begin{array}{c} Y_{\mathrm{TCC}}=582.48+176.97X_{1}+94.32X_{2}+67.59X_{3}+60.31X_{1}X_{2}\\ +35.70X_{1}X_{3}-39.47X_{2}X_{3}+24.34X_{1}^{2}-135.16X_{2}^{2}-23.34X_{3}^{2} \end{array}$$

### 3.3 Analysis of the significance of MAE parameters on TPC

**FIGURE 1:**
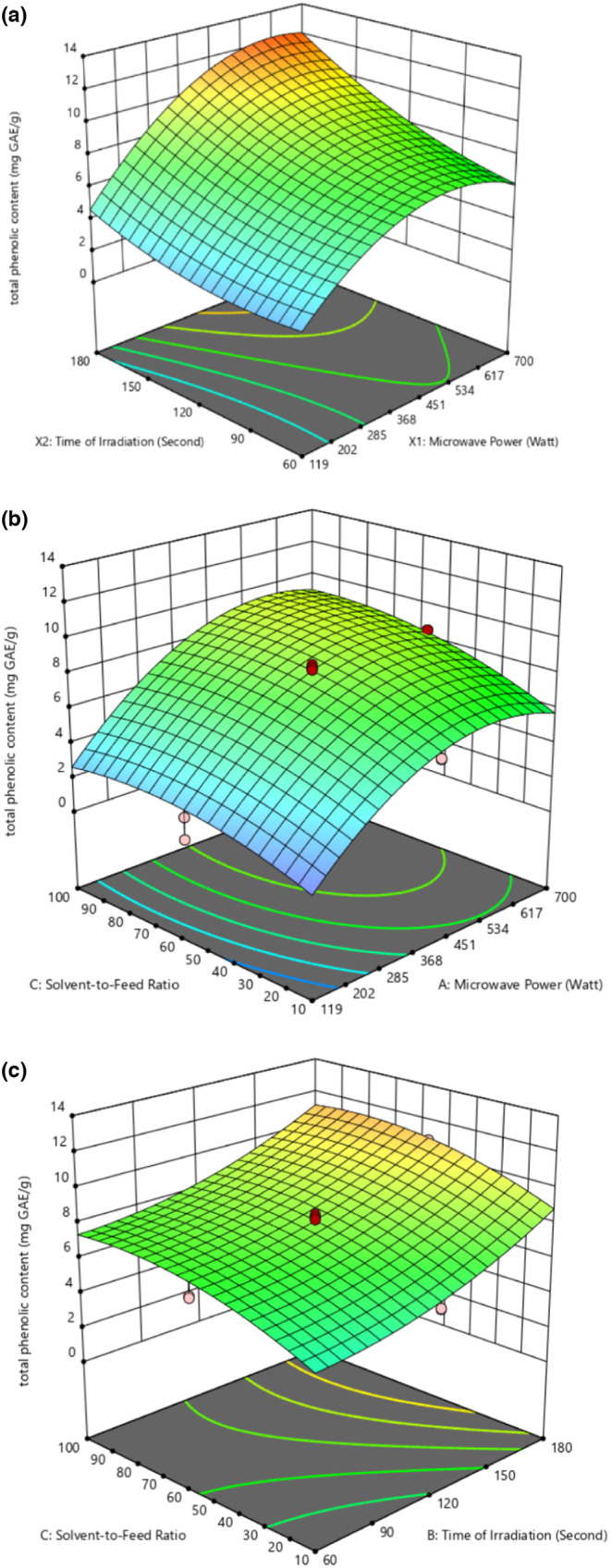
3D plot for total phenolic content presenting (a) the interaction effect of microwave power and time of irradiation. (b) Interaction effect of microwave power and solvent‐to‐feed ratio. (c) Interaction effect of time of irradiation and solvent‐to‐feed ratio.

The effects of MAE parameters and their mutual interactions on the TPC of the bioactive compounds from Sarawak *Liberica* sp. coffee pulp are presented in Table 3 and Figure 1a–c.

Figure 1a shows the response surface plot of TPC influenced by parameters *X*_1_ (microwave power) and *X*_2_ (time of irradiation) indicated as strongly significant with a *p*‐value of <.0001. The yield of TPC increased with the increase in microwave power from 119 to 534 W and time of irradiation from 60 to 180 s, respectively, then decreased as microwave power increased from 534 to 700 W. Similar results were obtained for MAE of polyphenols from spent espresso coffee grounds (Ranic et al., 2014), from lotus (*Nelumbo nucifera* Gaertn.) seeds (Zhang et al., 2012), and from avocado (*Persea americana* Mill.) seeds (Weremfo et al., 2020), in which the TPC increased with the increase in microwave power to a certain level, beyond which a drop in TPC was found. This phenomenon could be explained by the increased solubility of polyphenols at higher microwave power. The temperature of the extraction process is influenced by microwave power; an increase in power level will result in a higher temperature. High temperatures could break down chemical bonds or reduce intermolecular interaction, which increases the solubility of specific compounds and increases the number of extractable compounds in the solution (Xu et al., 2017). They can also soften plant tissue and increase solvent penetration into the sample, increasing the mass transfer rate (Dahmoune et al., 2013). Additionally, microwaves could break down the sample tissue and cell wall by creating a pressure differential between the interior and exterior of the plant cells or tissues. This results in the release of additional phenolic compounds. However, heat‐sensitive compounds, particularly phenolic compounds, can degrade at high temperatures, hence the decrease in the amount of TPC of bioactive compounds extracted from Sarawak *Liberica* sp. coffee pulp (Ali et al., 2018). As for the time of irradiation, the result was similar to Saifullah et al. (2020), who reported that longer MAE irradiation period resulted in larger yield of bioactive compounds extracted from lemon‐scented tee tree leaves. Time of irradiation represents the length of time the sample is submerged in the extraction solvent while being microwave irradiated. Longer radiation times resulted in more diffusion of the bioactive chemicals into the solvent, which increased extraction yields.

Figure 1b depicts the response surface plot of TPC influenced by parameters *X*_1_ (microwave power) and *X*_3_ (solvent‐to‐feed ratio) which is shown as significant (*p* = .0017). The extraction yield of TPC was likewise enhanced when the solvent‐to‐feed ratio increased from 10:1 to 90:1, but as the ratio continued to increase, the extraction yield of TPC gradually decreased. Higher solvent‐to‐feed ratio indicates that a greater amount of solvent was used. The results agree with the previous findings on traditional maceration extraction of phenolics from dried chokeberry reported by Ćujić et al. (2016); where phenolics increase when more solvent is used. This may be due to the saturation of phenolics in the solvent. When the amount of sample increased, more solvent is required to fully submerge the sample, hence minimizing the interaction between sample and solvent (Milutinović et al., 2014). In general, materials expand excessively when an extraction solvent is used in a bigger volume, which makes it simpler to break cell walls and extract polyphenols. However, in the presence of too much solvent, more energy will be absorbed by the solvent, and this reduces the material's capacity to absorb microwaves, which results in less effective extraction and a reduced polyphenol concentration in the extract (Maran et al., 2013). This could be the reason TPC yield decreased slightly with the further increase in solvent‐to‐feed ratio from 90:1 to 100:1.

The response surface plot of TPC as being influenced by the parameters *X*_2_ (time of irradiation) and *X*_3_ (solvent‐to‐feed ratio) is shown in Figure 1c, which is shown as nonsignificant with a *p*‐value of .7030. The *p*‐value was larger than .05, showing that *X*_2_ *X*_3_'s interaction effect on TPC yield was insignificant. This suggests that the solvent‐to‐feed ratio and time of irradiation each had an individual impact on the TPC yield but not their interaction effect.

### 3.4 Analysis of the significance of MAE parameters on TFC

**FIGURE 2:**
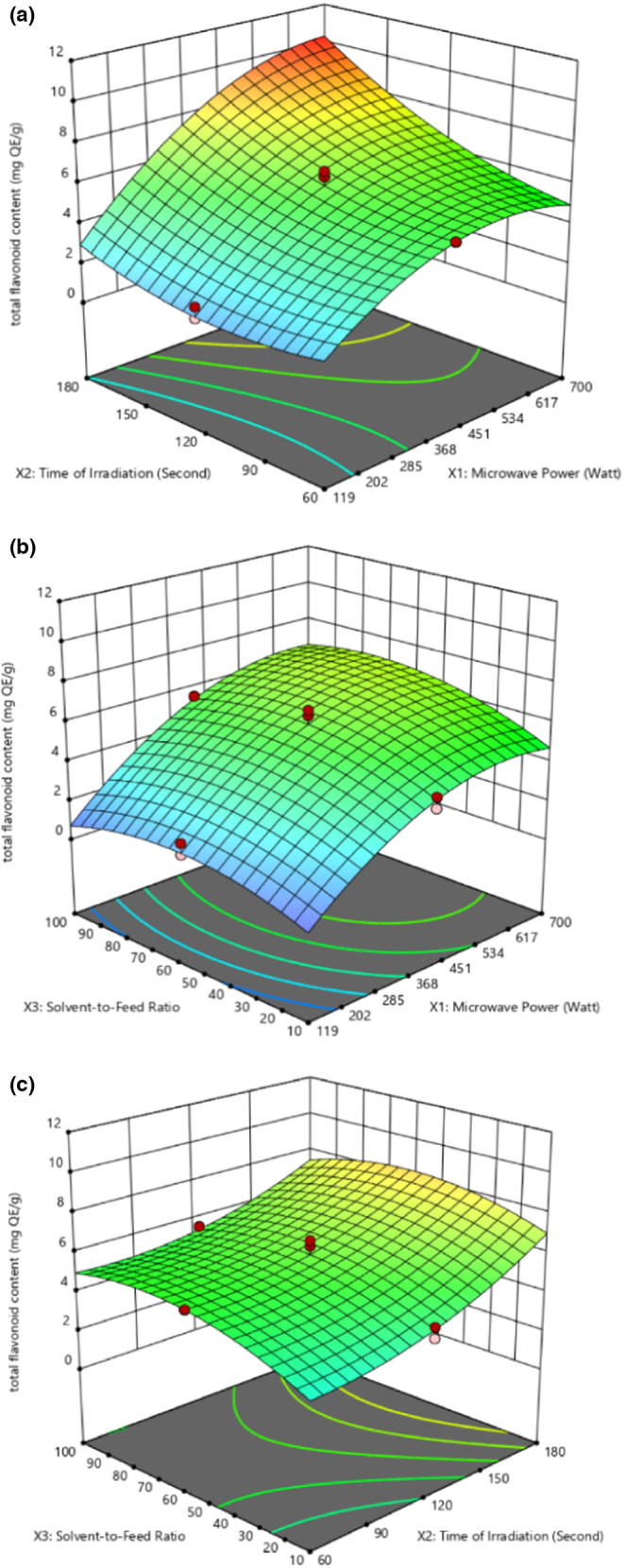
3D plot for total flavonoid content presenting (a) the interaction effect of microwave power and time of irradiation. (b) Interaction effect of microwave power and solvent‐to‐feed ratio. (c) Interaction effect of time of irradiation and solvent‐to‐feed ratio.

Table 3 and Figure 2a–c illustrate the effects of microwave power, time of irradiation, and solvent‐to‐feed ratio on the TFC of the bioactive compounds from Sarawak *Liberica* sp. coffee pulp.

As presented in Table 3, the linear effects of microwave power and time of irradiation and the interaction effect of microwave power and time of irradiation pair showed a strongly significant (*p* < .001) positive effect on TFC. In comparison, the quadratic terms of microwave power exhibited a strongly significant (*p* < .001) negative effect on TFC yield. Furthermore, the linear effect of solvent‐to‐feed ratio, the interaction effect of microwave power and solvent‐to‐feed ratio pair, and the quadratic terms of the time of irradiation showed a significant (*p* = .0016, *p* = .0402, and *p* = .0111, respectively) positive effect on TFC. In contrast, the quadratic terms of solvent‐to‐feed ratio showed a significant (*p* = .0016) negative effect on TFC. Nevertheless, the interaction effect of time of irradiation and solvent‐to‐feed ratio pair exhibited a nonsignificant (*p* = .0714) negative effect on TFC, which means that the pair does not have any effect on TFC.

An increase in microwave power, time of irradiation, and solvent‐to‐feed ratio from 119 to 617 W, 30 to 180 s, and 10:1 to 65:1, respectively, induces a rise in TFC. Further increasing microwave power and solvent‐to‐feed ratio from 617 to 700 W and 65:1 to 100:1 caused a decrease in TFC. The linear, interactive, and quadratic effects affecting TFC yield were very similar to those of TPC, which shows that the same factors that impacted the extraction of TPC also affected the extraction of TFC. It is reasonable to anticipate the observed outcomes, as flavonoids are a type of phenolic compound. Similar findings were reported by Woumbo et al. (2021), who noted that the responses of TPC and TFC were both influenced by the same effects for the MAE of polyphenols from soybean meal using the parameters (solvent/dry matter ratio, power, and time).

### 3.5 Analysis of the significance of MAE parameters on TCC

**FIGURE 3:**
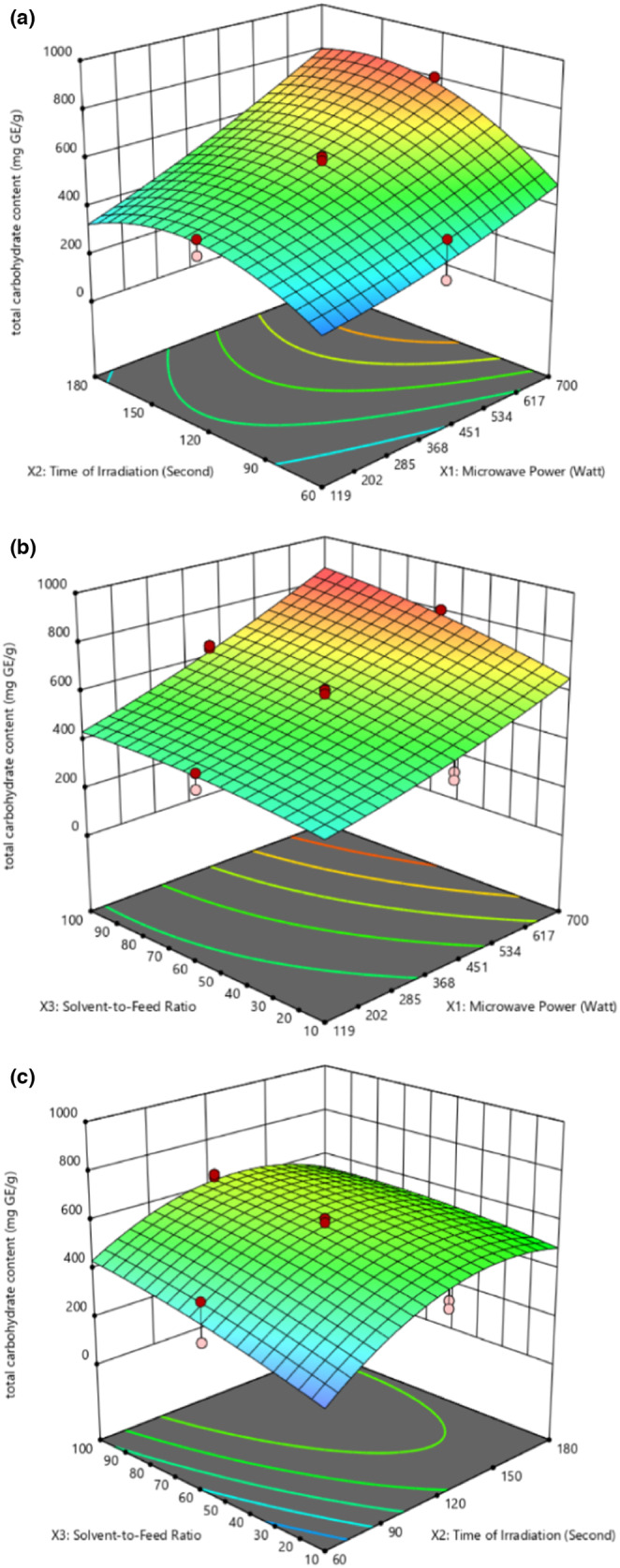
3D plot for total carbohydrate content presenting (a) the interaction effect of microwave power and time of irradiation. (b) Interaction effect of microwave power and solvent‐to‐feed ratio. (c) Interaction effect of time of irradiation and solvent‐to‐feed ratio.

As depicted in Figure 3a, the response surface plot of TCC influenced by parameters *X*_1_ (microwave power) and *X*_2_ (time of irradiation) is indicated as strongly significant with a *p*‐value of <.0001. The TCC yield increased with the microwave power and time of irradiation, and then, a decline was found as time of irradiation increased from 145 to 180 s. The result was consistent with Thirugnanasambandham et al. (2015) and Chen et al. (2007), who claimed that the increased microwave power and time of irradiation could accelerate the MAE of polysaccharide and the negligible effect was observed when the irradiation time exceeded a certain period. Radiation from microwave energy speeds up the lysis of the cell wall. Rapid transmission of magnetic energy to biomolecules increases the amount of power wasted inside the solvent, causing molecular mobility in the plant materials, and enhancing polysaccharide extraction effectiveness simultaneously (Maran et al., 2015). Along with increasing microwave power, dipole rotations would also rise, leading to power degeneration inside the reaction mixture. This causes the reaction mixture to heat up fast, increasing the polysaccharide yield. The TCC increased with time of irradiation, and reached its maximum yield at 145 s, then fell. The assumption is that the thermal accumulation in the extraction solution caused by the microwave energy's absorption accelerated the polysaccharide dissolution process into the solution for 145 s (Zheng et al., 2011). The deterioration of polysaccharides may occur, nevertheless, if they are exposed to microwave radiation over an extended period of time.

Figure 3b,c present the response surface plot of TCC influenced by parameters *X*_1_ *X*_3_ and *X*_2_ *X*_3_, respectively, indicated as significant with a *p*‐value of .0045 and .0020, respectively. From the figures, it is observed that the yield of TCC increased with increasing solvent‐to‐feed ratio. This can be explained by the fact that increasing solvent volume makes the compounds more mobile from plant matrix, which would account for the rise in the TCC at a certain solvent ratio. Moreover, increasing the solvent‐to‐feed ratio encouraged a rise in the extraction yields of bioactive compounds since a smaller amount of samples in the same volume of solvent might result in less saturation and thus boost overall extraction yields (Pham et al., 2017). Similar results were observed in the extraction of bioactive compounds from coffee pulp waste, where the extraction yields declined with the sample‐to‐solvent ratio increased (Tran et al., 2022a, 2022b).

### 3.6 Optimization and model validation

Based on the RSM analysis, an optimization procedure was performed to simultaneously maximize the yield of TPC, TFC, and TCC of the bioactive compounds from Sarawak *Liberica* sp. coffee pulp. The optimal conditions for the maximum yield of TPC, TFC, and TCC were determined to be a microwave power of 700 W, time of irradiation of 180 s, and solvent‐to‐feed ratio of 86.644:1 with desirability of 0.977. To demonstrate the models' validity in predicting the optimal conditions for the responses, experiments were performed under the optimized conditions, and the outcomes were compared with the predicted optimal yields. As shown in Table 5, the percentage difference between predicted and experimental values are less than 5%, and at 95% confidence level, the experimental values fell within the PI range generated by Design Expert Software, which confirms that the optimal conditions for the maximum yield of TPC, TFC, and TCC were validated successfully and can be used for further extraction of bioactive compounds from Sarawak *Liberica* sp. coffee pulp.

**TABLE 5:** Predicted and experimental values of the responses at optimum extraction conditions.

Responses	Predicted			Experimental	% Difference
	Mean	95% PI low	95% PI high		
TPC	12.77 ± 0.48	11.95	13.59	12.94 ± 2.25	1.31
TFC	10.19 ± 0.70	9.018	11.37	9.84 ± 0.38	3.49
TCC	836.60 ± 45.07	760.24	912.95	876.50 ± 64.15	4.55

*Note*: Results are presented as mean ± standard deviation of three replicates.

Abbreviations: PI, prediction interval; TCC, total carbohydrate content (mg GE/g); TFC, total flavonoid content (mg QE/g); TPC, total phenolic content (mg GAE/g).

### 3.7 Comparison of MAE with CME and SE

Based on the optimal extraction conditions (microwave power of 700 W, time of irradiation of 180 s, and solvent‐to‐feed ratio of 86.644:1) for maximizing the extraction yield, the best resulting MAE process was used to compare with CME and SE on the extraction efficiency of TPC, TFC, and TCC and the results are shown in Table 6. Table 7 provides information on the consumed sample, extraction time, and volume of solvent used in the preparation of the extracts using MAE, CME, and SE. The table presents the details for each technique, including the amount of sample used, extraction time, and volume of solvent employed in three replicates. In comparison with CME, MAE significantly (*p* < .05) improved the extraction of TPC, TFC, and TCC. Furthermore, as shown in Table 7, MAE requires less solvent and shorter extraction time as compared to CME. However, MAE was found to be less effective in extracting TPC, TFC, and TCC in comparison with SE. There was no significant difference (*p* > .05) between MAE and SE on the extraction efficiency of TPC, TFC, and TCC. It should be highlighted that compared to SE, MAE process took a much shorter time (3 min compared to 10 h). Besides, the solvent consumption for MAE (maximum 250 mL per run) was also less than for SE (350 mL).

**TABLE 6:** Extraction yields by three different extraction techniques.

Responses	Extraction technique		
	MAE	CME	SE
TPC	12.94 ± 2.25^b^	4.84 ± 0.17^a^	16.49 ± 0.75^b^
TFC	9.84 ± 0.38^b^	4.57 ± 0.32^a^	11.99 ± 1.35^c^
TCC	876.50 ± 64.15^b^	386.11 ± 19.03^a^	1042.42 ± 14.28^b^

*Note*: Results are presented as mean ± standard deviation of three replicates and the values in the same row with same superscript letter are not significantly different from each other (Tukey and Kruskal–Wallis tests, *p* > 0.05).

Abbreviations: CME, conventional maceration extraction; MAE, microwave‐assisted extraction; SE, Soxhlet extraction; TCC, total carbohydrate content (mg GE/g); TFC, total flavonoid content (mg QE/g); TPC, total phenolic content (mg GAE/g).

**TABLE 7:** Consumed sample, time, and solvent during the preparation by three different extraction techniques.

	Extraction technique					
	MAE		CME		SE	
Replicate	1	3	1	3	1	3
Amount of sample	2.5 g	7.5 g	5 g	15 g	5 g	15 g
Extraction time	3 min	9 min	16–24 h	16–24 h	10 h	30 h
Volume of solvent	250 mL	750 mL	350 mL	1050 mL	350 mL	1050 mL

Abbreviations: CME, conventional maceration extraction; MAE, microwave‐assisted extraction; SE, Soxhlet extraction.

### 3.8 Comparison of bioactive compounds’ yield with those reported in previous studies

A comparison of the TPC, TFC, and TCC yields of bioactive compounds extracted from different parts of various coffee species is presented in Table 8.

**TABLE 8:** Yield of total phenolic content, total flavonoid content, and total carbohydrate content in different parts of various coffee species.

Sample	Extraction method	Extraction solvent	Yield			References
			TPC	TFC	TCC	
*Liberica* sp. coffee pulp (present study)	Microwave‐assisted extraction	100% (v/v) methanol	12.94 ± 2.25^ab^	9.84 ± 0.38^b^	876.50 ± 64.15^c^	‐
*Arabica* sp. coffee bean	Aqueous extraction	70% (v/v) ethanol	35.39 ± 3.69^c^	‐	‐	Affonso et al. (2016)
*Arabica* sp. coffee silver skin	Aqueous extraction	50% (v/v) methanol	473.51 ± 56.70^e^	18.58 ± 2.47^c^	‐	Tan et al. (2017)
*Arabica* sp. coffee husk	Aqueous extraction	Hot water	‐	‐	88.88 ± 1.00^b^	Guglielmetti et al. (2019)
*Robusta* sp. coffee pulp	Microwave‐assisted extraction	50% (v/v) ethanol	46.72 ± 1.86^d^	36.44 ± 1.54^d^	‐	Tran et al. (2022a, 2022b)
*Robusta* sp. coffee pulp	Conventional maceration extraction	95% (v/v) ethanol	7.50 ± 0.50^a^	4.37 ± 0.22^a^	‐	Maxiselly et al. (2020)
*Robusta* sp. coffee bee pollen	Microwave‐assisted extraction	60% (v/v) ethanol	13.73 ± 0.37^b^	‐	‐	Quoc (2021)
*Robusta* sp. coffee silver skin	Aqueous extraction	Hot water	‐	‐	2.50 ± 0.30^a^	Guglielmetti et al. (2019)

*Note*: Results are presented as mean ± standard deviation of three replicates and the values in the same column with same superscript letter are not significantly different from each other (Tukey's test, *p* > .05).

Abbreviations: TCC, total carbohydrate content (mg GE/g); TFC, total flavonoid content (mg QE/g); TPC, total phenolic content (mg GAE/g).

Although the extraction yield of TPC and TFC from *Liberica* sp. coffee pulp in the present study was found to be lower compared to the other coffee species and parts listed in Table 8, it is important to note that the TPC and TFC yield of 12.94 ± 2.25 mg GAE/g and 9.84 ± 0.38 mg QE/g are still relatively high. Moreover, *Liberica* sp. coffee pulp is considered as a valuable source of bioactive compounds. This is especially important considering that *Liberica* sp. coffee has a unique flavor profile and is an important crop in Southeast Asia countries such as Malaysia. Therefore, it is worthwhile to explore the potential of *Liberica* sp. coffee pulp as a source of bioactive compounds for functional food and nutraceutical industries.

The extraction yield of TCC from *Liberica* sp. coffee pulp was significantly higher compared to the TCC yield of *Arabica* sp. coffee husks and *Robusta* sp. coffee silver skin as reported by Guglielmetti et al. (2019). This indicates that *Liberica* sp. coffee pulp can be a potential source of carbohydrates that can be used in various food applications. The high carbohydrate content in *Liberica* sp. coffee pulp can also make it a suitable substrate for the production of biofuels or biopolymers. Besides, the high TCC yield of *Liberica* sp. coffee pulp is consistent with the fact that coffee pulp is a by‐product of coffee production, which contains significant amounts of carbohydrates. The carbohydrate content of *Liberica* sp. coffee pulp can vary depending on various factors such as the ripeness of the fruit, the processing method, and the extraction technique used (Arena et al., 2023).

Table 9 shows the comparison of TPC, TFC, and TCC yields of bioactive compounds extracted from different parts of *Liberica* sp. coffee. Based on the results, the TPC yield from *Liberica* sp. coffee pulp in the present study was the lowest among all, and its TFC yield was slightly higher than those reported by Insanu et al. (2021). The slight difference in yields could be due to variations in the extraction conditions used in each study, such as solvent type, temperature, and duration of extraction. These variables can greatly influence the efficiency of extraction and the amount of bioactive compounds that can be extracted. The differences in coffee by‐products used in each study are important to consider because they can affect the composition and concentration of bioactive compounds in the extract. Different parts of the coffee plant may contain varying amounts of specific compounds (Pohl et al., 2013). The information on total carbohydrate content determination using phenol‐sulfuric method is lacking.

**TABLE 9:** Yield of total phenolic content, total flavonoid content, and total carbohydrate content in different parts of *Liberica* sp. coffee.

Sample	Extraction method	Extraction solvent	Yield			References
			TPC	TFC	TCC	
*Liberica* sp. coffee pulp (present study)	Microwave‐assisted extraction	100% (v/v) methanol	12.94 ± 2.25^a^	9.84 ± 0.38^b^	876.50 ± 64.15	‐
*Liberica* sp. coffee pulp	Conventional maceration extraction	95% (v/v) ethanol	19.25 ± 0.16^b^	18.19 ± 0.95^c^	‐	Maxiselly et al. (2020)
*Liberica* sp. coffee beans	Reflux extraction	100% (v/v) ethanol	22.59 ± 1.61^b^	3.36 ± 0.09^a^	‐	Insanu et al. (2021)
*Liberica* sp. coffee beans	Ultrasonic‐assisted extraction	60% (v/v) methanol	18.94 ± 0.06^b^	47.62 ± 0.05^d^	‐	Zainol et al. (2020)

*Note*: Results are presented as mean ± standard deviation of three replicates and the values in the same column with same superscript letter are not significantly different from each other (Tukey test, *p* > .05).

Abbreviations: TCC, total carbohydrate content (mg GE/g); TFC, total flavonoid content (mg QE/g); TPC, total phenolic content (mg GAE/g).

## 4 CONCLUSION

In conclusion, the optimal conditions for MAE of total phenolic, flavonoid, and carbohydrate content from Sarawak *Liberica* sp. coffee pulp were successfully optimized by RSM. Results suggest that three parameters, microwave power, time of irradiation, and solvent‐to‐feed ratio significantly enhanced the extraction yield of total phenolic, flavonoid, and carbohydrate content. The optimal MAE conditions generated by RSM are microwave power of 700 W, time of irradiation of 180 s, and solvent‐to‐feed ratio of 86.644:1. Under these conditions, the experimental results agreed with the predicted values; thus, the RSM models were verified. Comparison with CME confirmed the efficiency of MAE as it revealed the advantages of higher extraction yield within a shorter time of extraction. No significant difference was observed (*p* < .05) between MAE and SE on the extraction efficiency of TPC, TFC, and TCC. Nonetheless, MAE has the financial benefit of short extraction time with less solvent as compared to SE. The findings of this work offer fundamental understanding of MAE optimization for extraction of phytochemicals and may help with future research on bioactive compound extraction from coffee pulp for industrial purposes.

## AUTHOR CONTRIBUTIONS

**Joel Ching Jue Wong:** Conceptualization (lead); data curation (lead); formal analysis (lead); funding acquisition (equal); investigation (lead); methodology (lead); project administration (lead); resources (lead); software (lead); supervision (supporting); validation (lead); visualization (lead); writing – original draft (lead); writing – review and editing (lead). **Elexson Nillian:** Conceptualization (supporting); data curation (supporting); formal analysis (supporting); funding acquisition (supporting); investigation (supporting); methodology (supporting); project administration (supporting); resources (supporting); software (supporting); supervision (lead); validation (supporting); visualization (supporting).

## CONFLICT OF INTEREST STATEMENT

The authors declare that no potential conflict of interest exists.

## Data Availability

The data that support the findings of this study are available from the corresponding author upon reasonable request.
